# Expert consensus on the clinical application of recombinant adenovirus human *p53* for head and neck cancers

**DOI:** 10.1038/s41368-021-00145-1

**Published:** 2021-11-16

**Authors:** Yi Li, Wei Guo, Xiuqin Li, Jianguo Zhang, Moyi Sun, Zhangui Tang, Wei Ran, Kai Yang, Guilin Huang, Longjiang Li

**Affiliations:** 1 State Key Laboratory of Oral Diseases & National Clinical Research Center for Oral Diseases & Department of Head and Neck Oncology, West China Hospital of Stomatology, Sichuan University, Chengdu, China; 2 Department of Oromaxillofacial Head and Neck Oncology, Shanghai Ninth People’s Hospital, College of Stomatology, Shanghai Jiao Tong University School of Medicine, Shanghai, China; 3 Department of Obstetrics and Gynecology, Shengjing Hospital China Medical University, Shenyang, China; 4 Department of Oral and Maxillofacial Surgery, School and Hospital of Stomatology, Peking University, Beijing, China; 5 Department of Oral and Maxillofacial Surgery, The Third Affiliated Hospital, Air Force Medical University, Xi’an, China; 6 Department of Oral and Maxillofacial Surgery, Xiangya School of Stomatology, Central South University, Changsha, China; 7 Department of Oral and Maxillofacial Surgery, First Affiliated Hospital of Sun Yat-sen University, Guangzhou, China; 8 Department of Oral and Maxillofacial Surgery, The First Affiliated Hospital of Chongqing Medical University, Chongqing, China; 9 Department of Oral and Maxillofacial Surgery, Stomatological Hospital, Zunyi Medical University, Zunyi, China

**Keywords:** Cancer therapy, Head and neck cancer

## Abstract

The first gene therapy product, recombinant adenovirus human *p53* (rAd-*p53*), has been approved by CFDA since 2013. During these years, most of the clinical trials and the relevant basic research were carried out by Chinese oncologists. Gendicine was proved to be a safe and promising gene therapy drug for patients who suffered from head and neck squamous cell carcinoma (HNSCC). The basic therapeutic theories of gene therapy were totally different from the traditional ones, such as surgeries or radio- and chemotherapy, and the evaluation of treatment outcomes should also be changed simultaneously. However, there still existed a lot of misunderstandings about gene therapy, which resulted in improper administration, insufficient dosage calculation, and treatment cycles, and the treatment outcomes were unsatisfactory, especially for inexperienced oncologists or hospitals. Therefore, we will provide some practical guidance here on the gene therapy of rAd-*p53* based on our previous research and experience, which focused on the basic theories and clinical issues, to answer the questions arising during the clinical of gene therapy and to accelerate the development of gene therapy for the benefit of patients bearing malignant tumors.

## Introduction

Deoxyribonucleic acid (DNA) is a fundamental genetic substance. It is universally acknowledged that most diseases are related to genetic disorders, especially solid malignancies. If the gene transfer can be efficiently conducted to repair the disorders, it will add a new dimension to the treatment of diseases. Therefore, the gene transfer has been paid a great deal of attention. Gene therapy works by replacing defective genes with normal ones and therapeutic drugs are usually administered through viral vectors or genetically engineered microorganisms.^1^ Recombinant Adenovirus human *p53* (rAd-*p53*) (Gendicine™) was approved by the China Food and Drug Administration (FDA) in 2003, becoming a worldwide first-line drug for the treatment of head and neck squamous cell carcinoma (HNSCC).^2^ However, a similar virus (Advexin, Introgen) failed to receive US FDA approval in 2008. Another clinical trial has recently been conducted using adenovirus expressing *p53* (Ad-*p53*, MultiVir) in combination with immune checkpoint inhibitors in recurrent or metastatic head and neck cancer (www.clinicaltrials.gov, NCT03544723).^3^ To date, a great many clinical trials have proved that it is effective enough as a wide-spectrum anticancer agent. From 2000 to 2012, most of the gene therapy-related clinical trials were performed in China, and Chinese researchers and physicians had accumulated a wealth of experience in nearly 20-year clinical practice. Ultimately, it turned out that gene therapy is a safe and promising approach for HNSCC depending on optimal treatment design and drug administration.^4^ Therefore, we will provide some practical guidance on the clinical application of gene therapy for head and neck cancers.

## The basic function of *P53* gene

**Fig. 1:**
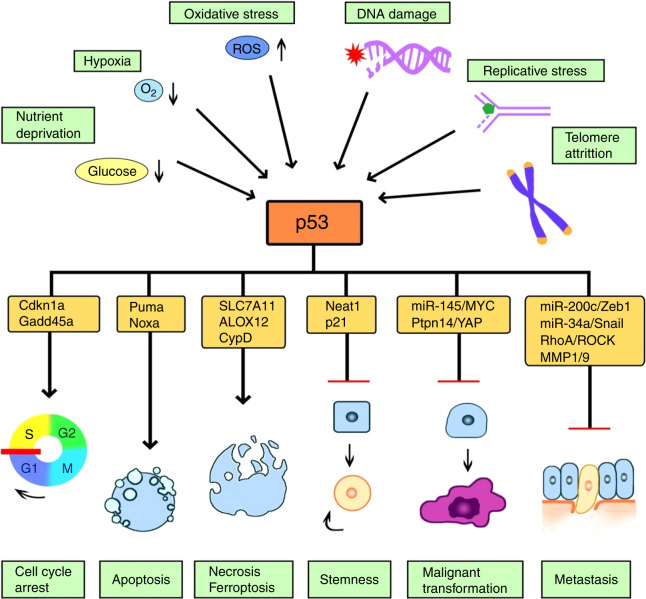
Biological functions of p53

*P53* is closely associated with multifaceted biological functions, including but not limited to DNA replication, modification and repair, cell cycle regulation, cell-fate determination, angiogenesis, anti-infection and immunity.^5,6^ P53 plays an important part in cell cycle by regulating cell cycle checkpoints of G1/S, S, and G2/M phases to bring a halt to cell cycle progression and DNA replication, while allowing DNA repair. Both cell cycle arrest and cell apoptosis are p53-mediated canonical tumor-suppressive effects.^7,8^ P53-mediated apoptosis is realized by activating the caspase cascades and shifting the balance of the Bcl-2 family through the mitochondria intrinsic pathway.^9^ Besides apoptosis, recent studies discovered p53 suppresses cystine uptake and sensitizes cells to ferroptosis via inhibiting the expression of a key component of the cystine/glutamate antiporter, SLC7A11.^10^ It was also found that p53 inhibited cancer metastasis by transactivating miR-200c and miR-34a to interfere the key epithelial-mesenchymal transition drivers to abrogate this process.^11,12^ P53 also restrains c-Myc signaling and Yap-Hippo pathway via transactivating miR-145 and Ptpn14, respectively, to repress cancer progression.^13,14^ Moreover, p53 is found to regulate autophagy by activating autophagy-related genes (such as *AMPK*, *DRAM*, *SESN1*, and *SESN2*), thus inhibiting tumor growth and proliferation.^15^ In addition, p53 may play indirect roles in regulating the downstream functional proteins as a tumor suppressor by restraining stemness via miR-34a and Neat1,^16^ inhibiting angiogenesis,^17^ mediating cellular senescence,^18^ generating a bystander effect,^19^ modulating tumor microenvironment,^20^ inducing immune responses^21^, and so on. The basic mechanisms underlying these canonical and non-canonical functions of p53 are briefly summarized below (Fig. 1).

## Expert group

The famous head and neck oncologists from the committee of integrative medicine of oral and maxillofacial oncology of the Chinese Anti-Cancer Association were invited to discuss and conduct this expert consensus. The ten participants were from nine different institutions all over China and all of them worked clinically. The participants’ backgrounds included head and neck surgery and medical oncology. The participants had an average 23.9 years of research experience in the field of clinical studies of head and neck oncology or related areas since their doctoral degree. A workgroup of two members (L.J.L. and Y.L.) prepared the draft and an online meeting was convoked to invite all the attendees to discuss the contents and form the final version of the present expert consensus.

## Gene transfer

Gene transfer refers to a biomedical technology through which normal and therapeutic genes can be transferred into target cells in order to correct genetic disorders and consequently achieve therapeutic purposes.^22^ One of the correcting methods is the in situ reparation of genetic defects, another is the replacement of tumor suppressor genes with normal and functional genes from the patient’s genome. Different from traditional therapeutic methods, gene therapy is targeted at abnormal genes. It is verified that several approaches of gene therapy have been successfully performed to transfer normal genes into target cells, including biological, physical and chemical transfer.^23^ Further adenoviruses are shown to be the most common vectors of gene transfer.^24^ With the development of biotechnologies and a growing understanding of genetic diseases, a growing number of types of gene therapy have flourished, including gene correction, gene replacement, gene augmentation, gene deactivation, suicide gene, immunotherapy, and drug resistance.^25^

## The selection of target genes and vector construction

*P53* may be an ideal target of gene transfer, as the *p53* gene plays a vital part in anti-tumor responses in normal cells and its mutations are frequently observed in solid malignancies. It is the momentous role of *p53* in the occurrence and development of malignancies that makes it a key gene of anti-tumor gene therapy. Exactly taking advantage of the core role of the *p53* gene, Chinese researchers produced rAd-*p53*, which transferred a single gene into tumor cells and then gave rise to a series of anti-tumor biological capabilities so as to achieve the treatment of malignant tumors. rAd-*p53* restricts its viral infectivity to a single cell cycle and the adenoviral genome does not fuse with host genomic DNA nor does it impair normal cell.^26^

The rAd-*p53* is a recombinant human serotype 5 adenovirus where a human wild-type *p53* expression cassette takes the place of E1 region. rAd-*p53* is generated by proprietary production cell lines grown in bioreactors. rAd-*p53* generated by the bioreactor is further treated and purified by chromatography to obtain the injection reagent.^27^ Recombinant adenovirus is the most widely used viral vector at present, by virtue of its favorable characteristics for gene therapy, for instance, high gene transfer efficiency, large gene carrying capacity, and mild cytotoxicity.^28^

## Indications and contraindications of rAd-*p53* in the treatment of HNSCC

### Indications

Numerous studies have confirmed that *p53* mutations occurred in more than 70% of HNSCC and in more than 50% of other malignancies throughout the body. Therefore, *p53* gene mutation is viewed as a common phenomenon in human solid malignancies. All cancer patients with *p53* gene mutations detected before treatment (concurrent with the biopsy diagnosis) are in the indication group of rAd-*p53* treatment. It is commonly used in patients with locally advanced stage, metastasis, or/and recurrence, in combination with radiotherapy, thermotherapy, chemotherapy, biological therapy, and other types of therapies.

### Contraindications

Significant cardiac, hepatic, renal, pulmonary, or other major organ failures; severe myelosuppression and severe coagulation dysfunction that cannot be corrected; uncontrolled hypertension and diabetes mellitus; neuropsychiatric or immune system disorders; pregnant or lactating women; Eastern Cooperative Oncology Group (ECOG) physical status score ≥ 3 or Karnofsky behavioral status score < 70; expected survival time < 3 months; other patients who are not suitable for rAd-p53 therapy.

## Administration methods and therapeutic schemes

### Dosage calculation

The efficient transduction of gene therapy reagents relies on a high multiplicity of infection (MOI), which is defined as the proportion of agents to the target cells. In other words, the virus particles are supposed to be adequate enough to transfer into target cells. However, it is of vital significance to obtain an optimal MOI in the administration of gene therapeutic drugs, for an excess of virus particles can result in severe hepatic damage and even individual death.^29^ Conventional dose determination methods utilized in chemotherapy, which depend on the body surface area or body weight, may be inappropriate for gene therapy. The basic elements of dosage calculation of gene therapy reagents involve malignant cell quantity calculated from the tumor volume and the mean cell density in the nidus, as well as the routes of administration. The tumor volume was measured by computed tomography (CT) or magnetic resonance imaging (MRI) before therapy, the total number of cells per cubic centimeter of a solid malignant tumor is 1 × 10^9^, the MOI for the one-time successful transduction of 80% of tumor cells is 100, and the dosage calculation formula of local administration is as follows.^30^

$${{{\mathrm{Dosage}}}}\,{{{\mathrm{per}}}}\,{{{\mathrm{administration}}}}\left( {{{{\mathrm{vp}}}}} \right) = {{{\mathrm{tumor}}}}\,{{{\mathrm{volume}}}}\left( {{{{\mathrm{cm}}}}^3} \right) \times {{{\mathrm{mean}}}}\,{{{\mathrm{cell}}}}\,{{{\mathrm{density}}}}\left( {10^9} \right) \times {{{\mathrm{MOI}}}}\left( {100} \right)$$

### Routes of administration

To ensure the dose concentration in the tumor area, it is ideal to increase the therapeutic dose of gene therapy through local administration. Human rAd-*p53* is usually administered locally, intra-tumoral injection, intra-arterial infusion, and intracavitary perfusion included.^31^

#### Intra-tumoral injection

The most important point of intra-tumoral injection lies in the three dimensions of tumors, which means that we should not only divide a tumor into nine gridded surfaces but also conduct a zoning design considering the depth of invasion. Therefore, CT or MRI images should be carefully read and fully comprehended before injection. Three-dimensional reconstruction is of great help to formulate treatment plans. Superficial tumors can be injected directly, while ultrasound-guided injection can be utilized for deep tumors. According to the size of tumors, the preparation should be diluted with an appropriate amount of 0.9% saline before injection to ensure that the amount of virus particles per milliliter is not <1 × 10^11^ vp.

Prior to multipoint intra-tumoral injections, local block anesthesia is advised for eliminating pain in the local area. According to the optimal infection titer of rAd-*p53* (MOI = 100) and the single maximal toleration dosage, the injection specification is determined as 1 × 10^11^ vp/cm^3^/ml, which means one injection site for 1 cm^3^ and 1 ml rAd-*p53* solution for one site.^32^ The injection needle should be inserted vertically into the tumor nidus. The injection should cover the entire tumor and the para-cancer tissues within about 0.5 cm.

### Arterial infusion

Each anatomical area of the head and neck is supplied by a well-defined branch system of the external carotid artery, which is quite conducive to the utilization of arterial drug delivery. Prior to administration, a retrograde arterial cannula of the superficial temporal artery is performed under local anesthesia. The catheter should be put into the artery supplying the region to be treated and the drug delivery equipment should be placed subcutaneously in the temporal area.^33^ The dosage is estimated depending on the tumor volume and the preparation was diluted to 30 ml with 0.9% saline, and the infusion was controlled by a micropump within 30 min to ensure the duration of transduction.

#### Other routes of administration

Human rAd-*p53* is mainly used for the treatment of head and neck malignancies, through the above-mentioned intra-tumoral injection and arterial infusion. As for the treatment of malignancies in other parts, there is an expanded indication. Clinical experience showed that interventional administration combined with hepatic artery embolization was suitable for the treatment of hepatocellular carcinoma, and intraperitoneal or thoracic perfusion is suitable for the treatment of metastatic carcinoma in the abdominal or thoracic cavity, malignant pleural effusion, or ascites. Notably, intravenous administration should not be adopted, as the dosage has to be increased (the maximum single dosage of Gendicine should not exceed 6 × 10^12^ vp) to achieve the therapeutic outcomes on account of the broad distribution of products. rAd-*p53* lacks the targeting ability to the tumor cells and the higher dosage will not increase the concentration significantly in the tumor focuses, but may lead to serious complications, such as liver failure or damage of pulmonary function, which might be caused by the aggregation of rAd-*p53* in the lung and liver during intravenous administration.

### Administration cycles

**Fig. 2:**
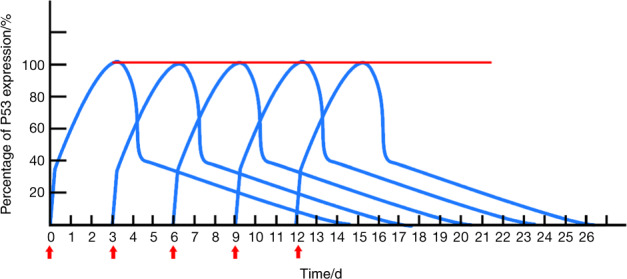
Diagram of *p53* expression in the cancer cells during the periodical transferring of exogenous *wt-p53* gene. Note: “the red line” indicating the continuous high expression of *p53* proteins; “the red arrows” indicating the time points for the administrations of rAd-*p53*, the treatment cycles are 3 days between each administration point

Studies have demonstrated that WT-p53 and its downstream factors peak at 72 h after successful transduction and then decline.^26,30^ Due to the rapid depletion of exogenous genes, the effects of gene therapy do not last long enough to maintain high levels of functional proteins. Therefore, transduction should be repeated every 72 h after the first administration, to keep the continuous expression of *p53* to guarantee practical self-repair of tumor cells. Frequent administration is indispensable in gene therapy, as it is difficult to effectively and completely modify all malignant cells just in one transduction, and thus a series of cycles are necessary for accomplishing the aim. Nevertheless, the association between the expression and function of target genes remains unclear. Theoretically, it should be ensured that target genes are sustainedly and highly expressed in cancer cells to impose an influence on the cytogenetic material. As a consequence, the treatment of human rAd-*p53* for HNSCC should include a regimen of at least five cycles of transduction to guarantee effective outcomes (Fig. 2).

### Therapeutic schemes

#### In combination with radiotherapy and chemotherapy

Gene therapy can play a vital role in combination therapies.^34^ Cells have a precise regulatory system in response to radiation-induced or chemical-induced DNA damage, and the *p53* gene plays a crucial part in the DNA repair system.^35,36^ The p53-dependent gene activation profile can drive tumor cells towards apoptosis or cell cycle arrest, and this pattern relies on the posttranslational modification of p53.^37,38^

Based on these mechanisms, it is possible to kill tumor cells to the maximum extent and at the same time protecting normal ones. Hence, the *p53* gene can be regarded as a potential therapeutic target to reduce adverse reactions.^39,40^ Besides, p53 influences the chemosensitivity of tumor cells through senescence and bystander effects, which imply that some tumor cells not directly treated with chemotherapy or radiotherapy can be killed or damaged through the diffusion of soluble pro-death factors in the target cells. Consistent with radiotherapy, chemotherapy drugs are primarily targeted at damaging DNA.^41,42^ In both chemotherapy and radiotherapy, the p53-dependent signaling pathway activated by DNA damage exhibits similarities and the *WT-p53* gene plays a crucial part in the induction of apoptosis. Therefore, it is of great importance to combine chemoradiotherapy with rAd-*p53* gene therapy to repair damaged DNA and lessen the therapeutic resistance. In addition, WT-p53 protein is able to attenuate the poisonousness of chemotherapy agents through some ways: (1) the interaction between p53 protein and DNA helicase; (2) p53 increases ribonuclease reductase; and/or (3) 3′→5′ exonuclease activity of p53 protein.^43,44^ It is indicated that the combination application of rAd-*p53* significantly facilitates the inhibition of chemotherapy agents on the proliferation and apoptosis of malignant cells.^21,45^

The mechanisms of chemoradiotherapy and gene therapy are fairly distinct and these therapeutic strategies can complement each other. In clinical practice, the first step is the administration of gene therapy to reconstruct the p53 pathway and to concentrate cancer cells in the G1 phase, which constitutes the so-called “cell preparation,” then followed by chemotherapy and/or radiotherapy starting on day 3 after the first administration of human rAd-*p53*. With cisplatin and 5-Fu or docetaxel, and cisplatin and 5-Fu as chemotherapy regimens and external irradiation for radiotherapy, gene therapy drugs still keep administered in cycles during chemotherapy and/or radiotherapy. Such a procedural therapeutic scheme allows achieving better results, while minimizing treatment resistance.^46,47^

#### In combination with thermotherapy

As thermotherapy has become an adjuvant treatment for cancers, the combination of gene therapy with thermotherapy constitutes an effective therapeutic strategy. The transduction of the normal *p53* gene using a viral vector can inhibit and reverse the malignant phenotype of tumors and induce thermal sensitization or radiosensitization, which is a novel strategy for converting a heat-resistant or radiation-resistant phenotype into a thermosensitive or radiosensitive phenotype.^48^ In clinical practice, after two cycles of the intra-tumoral injection of rAd-*p53*, superficial tumors were suggested to be treated with a 915 MHz microwave device at 43 °C–44 °C for 1 h of thermotherapy 2 days a week, and a 41 MHz radiofrequency machine at 42 °C–43 °C for 1 h of thermotherapy 2 days a week for deep tumors. The gene therapy drugs were still administered periodically during treatment and radiotherapy can also join in the combination treatment.

#### Clinical trials

For those patients with advanced tumors and mutant *p53* gene detected, who have been clinically treated with a variety of therapies that are proved to be ineffective or are in need of alleviated symptoms, they can voluntarily participate in clinical trials for the expanded indications of rAd-*p53*. It is believed that it can seek more therapeutic benefits through combination therapies including radiotherapy, chemotherapy, targeted drugs, immune agents, and others. More than 16 clinical trials of rAd-*p53* have been performed during the last two decades in China. The pathologic types containing HNSCC,^30,49–51^ non-small-cell lung cancer,^52,53^ nasopharyngeal carcinoma,^54–56^ hepatic cell carcinoma^57–61^, malignant gliomas^62^, and ovarian carcinoma,^63^ etc. These clinical trials demonstrated that rAd-*p53* treatment groups exhibit better overall survival rates than control groups.

## Efficacy evaluation and adverse effects

### Efficacy evaluation

The main objective of gene therapy completely differs from that of conventional therapies. The latter aims to get rid of the tumor by various means, such as surgery, chemotherapy, and radiotherapy. The main aim of chemoradiotherapy is to induce apoptosis by breaking DNA single and double strands, and destroying bases, and on the other hand, surgery is conducted to entirely eradicate tumor itself.^64,65^ Based on these therapeutic principles of conventional therapies, an international group collaborated to publish the Response Evaluation Criteria in Solid Tumors (RECIST) to evaluate treatment efficacy, which described when a tumor responds, when it remains stable, and when it progresses during the course of treatment. Comparing with the original evaluations, RECIST 1.1 is an evidence-based one, which evaluates more target lesions, highlights the necessity of the assurance of therapeutic effects, and updates the evaluation methods of lymph nodes. As neither the RECIST nor RECIST 1.1 can evaluate the effect of immune therapy appropriately, iRECIST has been developed.^66,67^

The fundamental goal of gene therapy is to turn the correct defective genes, which means to repair the genetic mutation or deletion, to bring an end to the immortal cancer cells, and to return them to their normal programmed cell death state. Because of the theoretical differences from the conventional therapies and immunotherapy, the evaluation of gene therapy should be different. It has been revealed that neither the RECIST, RECIST 1.1, nor the iRECIST was suitable for the therapeutic assessment. Treated with gene therapy combined with chemotherapy and/or radiotherapy, many patients are satisfied with good therapeutic effects. Although cancer foci can still be observed on CT or MRI, fewer subjective symptoms are manifested and overall survival is remarkably improved, and it is reasonably believed that the patients can live with tumors for a longer time.^30^ These better results are closely related to the principle of gene therapy, which is to replace tumor suppressor genes with normal and functional genes, rather than kill the entire tumor cell. Therefore, in our clinical practice, we cannot observe a rapid reduction in the size of the primary or metastatic cancer foci at the early stage of the combination therapies, but patients’ symptoms greatly improved, which is not same as what is observed in the conventional treatment strategies. Based on the above experience, we reckon that the RECIST is not appropriate as the evaluation criteria for gene therapy and thus new efficacy evaluation guidelines should be developed for future clinical trials of gene therapy.

With the *p53* gene transfected into tumor cells, they are “civilized” and display weakened malignant biological capabilities. Hence, human rAd-*p53* treatment for malignant tumors is expected to be the most promising therapeutic approach to achieve long-term “live with tumor.”

### Adverse effects

The clinical application of human rAd-*p53* is rather safe, with few serious adverse effects. The most common treatment-related adverse reaction is transient flu-like symptoms, clinically manifested by muscle soreness and fever (body temperature around 38 °C) on the day of administration. The rise in temperature is self-limited and the symptoms start to subside the next day without treatment. In cases of persistent high fever, physical cooling (alcohol baths) or medication (Nimesulide 25–50 mg) can be applied to reduce fever.

A rare complication is platelet crisis, featured by a sharp drop in platelet count below 30 × 10^9^/L after administration and severe spontaneous bleeding. It should be treated promptly by blood transfusion, immunosuppressants, and hemostatic drugs, otherwise the patient’s life will be endangered. Platelet crisis may be associated with immune dysfunction of gene therapy vectors, which can be effectively avoided by strictly grasping contraindications and carefully assessing patients’ biochemical indexes and ECOG physical status before treatment.

## Conclusions

At present, rAd-*p53*, a novel gene therapy reagent, has been widely applied to treat HNSCC, and been proved safe and effective in clinical practice. Based on gene therapy-related clinical trials and specialist experience, we provided and summarized the guidance on the clinical application of rAd-*p53* for better facilitate the management of solid malignancies.

Both indications and contraindications of rAd-*p53* are explicitly stated in this guidance, yet the former is not quite absolute. There are two major routes of administration: local intra-tumoral injection and arterial infusion (via superficial temporal artery), whereas intravenous administration should not be adopted. Moreover, the recommended dosage per administration of local intra-tumoral injection should be no less than 1 × 10^11^ vp/cm^3^/ml. According to the half-life of rAd-*p53*, the whole treatment scheme should contain at least 5 administration cycles (every 72 h) in order to attain favorable outcomes. In addition, when the clinical application of rAd-*p53* as the gene therapy drug is in combination with radiotherapy, chemotherapy, or thermotherapy, it is possible to kill tumor cells to the maximum extent while preserving normal tissues, thus enhancing the therapeutic efficacy. Nevertheless, the evaluation of gene therapy does not coincide with that of traditional therapeutic strategies. Therefore, the future direction may fall on establishing and developing a novel evaluation system for the efficacy and safety of gene therapy.
